# The Transjugular Intrahepatic Portosystemic Shunt in the Treatment of Portal Hypertension: Current Status

**DOI:** 10.1155/2012/167868

**Published:** 2012-07-19

**Authors:** Gilles Pomier-Layrargues, Louis Bouchard, Michel Lafortune, Julien Bissonnette, Dave Guérette, Pierre Perreault

**Affiliations:** ^1^Liver Unit, Centre Hospitalier de l'Université de Montréal, Montreal, QC, Canada H2X 3J4; ^2^Department of Radiology, Centre Hospitalier de l'Université de Montréal, Montreal, QC, Canada

## Abstract

The transjugular intrahepatic portosystemic shunt (TIPS) represents a major advance in the treatment of complications of portal hypertension. Technical improvements and increased experience over the past 24 years led to improved clinical results and a better definition of the indications for TIPS. Randomized clinical trials indicate that the TIPS procedure is not a first-line therapy for variceal bleeding, but can be used when medical treatment fails, both in the acute situation or to prevent variceal rebleeding. The role of TIPS to treat refractory ascites is probably more justified to improve the quality of life rather than to improve survival, except for patients with preserved liver function. It can be helpful for hepatic hydrothorax and can reverse hepatorenal syndrome in selected cases. It is a good treatment for Budd Chiari syndrome uncontrollable by medical treatment. Careful selection of patients is mandatory before TIPS, and clinical followup is essential to detect and treat complications that may result from TIPS stenosis (which can be prevented by using covered stents) and chronic encephalopathy (which may in severe cases justify reduction or occlusion of the shunt). A multidisciplinary approach, including the resources for liver transplantation, is always required to treat these patients.

## 1. Introduction

Portal hypertension is associated with severe and often life-threatening complications. Increased intrahepatic resistance results in increasing splanchnic blood flow and development of venous collaterals, which may bleed, and also causes splenomegaly. A hyperdynamic circulation develops with an increased cardiac output and a decrease in systemic vascular resistance. Pooling of splanchnic blood may result in a systemic hypovolemia, which can trigger activation of vasoactive systems, mainly vasoconstrictors. This in turn may lead to sodium retention, ascites, and ultimately hepatorenal syndrome [1]. The correction of severe portal hypertension by portacaval shunt surgery has been used for many years, but the morbidity and mortality were high. Moreover, this technique was contraindicated in the presence of liver failure.

The transjugular intrahepatic portosystemic shunt (TIPS) was used for the first time by Rösch et al. in 1969 [2] in dogs and in a cirrhotic patient by Colapinto in 1982 [3]. This treatment was aimed at nonsurgically decreasing portal hypertension. Originally, a tract was created by balloon dilatation of the parenchyma between the hepatic vein and the portal vein after transjugular portal vein catheterization. Unfortunately, this communication closed within days after the procedure. In 1989, the first case of TIPS created with a metallic stent was published by Rössle et al. [4]. This technical advance allowed good long-term patency of the shunt.

Many papers were published in the following years, which led to technical improvements and definition of the best indications for this promising treatment of complications of portal hypertension [5].

In the present paper, technical aspects of this procedure will be described, and the current indications based on the existing literature will be discussed. Contraindications (absolute and relative) will be reported and the potential complications following the TIPS procedure as well as their treatment will be mentioned.

## 2. Technical Aspects

TIPS is a hemodynamic equivalent of a side-to-side small diameter surgical portacaval shunt. The experience gained over the last 20 years allows thorough evaluation of the complications of this technique and of its contraindications and indications [5, 6].

**Figure 1:**
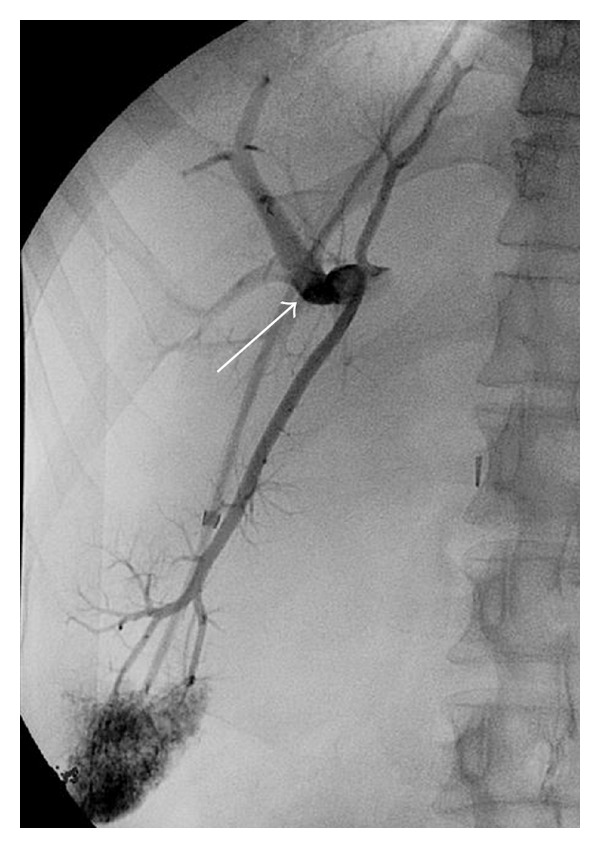
Wedged hepatic venography allowing intrahepatic portal vein localisation (arrow).

**Figure 2:**
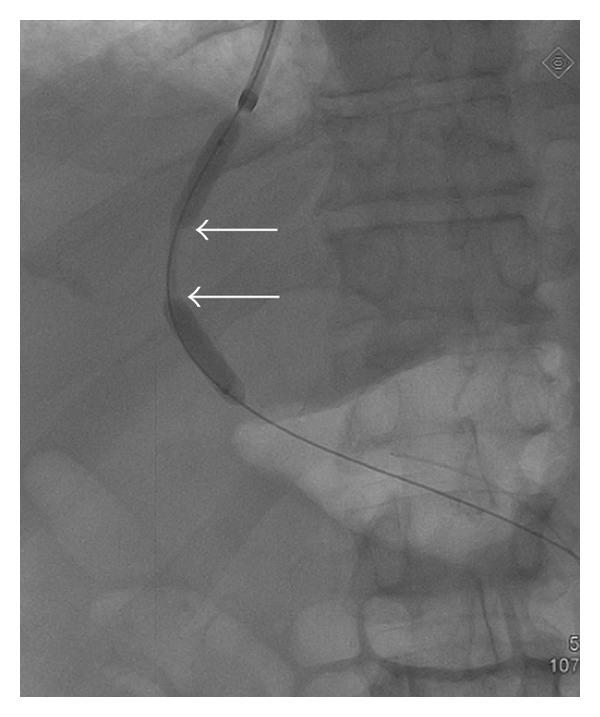
Tract dilatation using a 10 mm angioplasty balloon catheter. The narrowed part of the balloon is in the intraparenchymal part of the tract (arrows).

**Figure 3:**
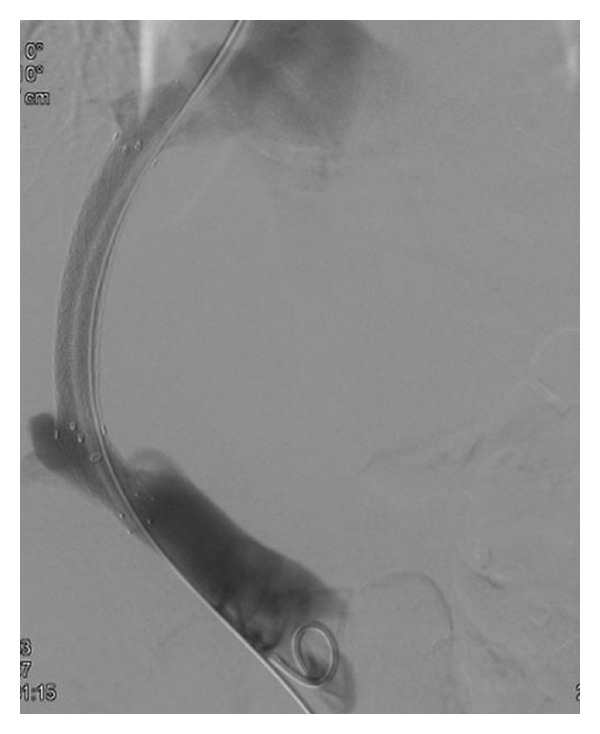
TIPS made with PTFE-covered stents between portal and right hepatic veins.

This technique is preferably done under general anesthesia [7] but can be performed with deep sedation (particularly for emergency cases). Antibiotic prophylaxis is given even if the literature has not proven the usefulness of this approach, and coagulation defects are corrected before the procedure. After puncture of the jugular vein (most often the right jugular vein) under echographic guidance, a catheter is introduced into one hepatic vein and wedged in the liver parenchyma. Gentle injection of dye allows the retrograde visualisation of intrahepatic portal vein branches [8] (Figure 1). The intrahepatic portal vein then is entered with a modified Ross needle (Cook Medical, Bloomington, IN, USA). CO_2_ can be used in patients with renal function impairment to avoid dye nephrotoxicity. Several TIPS sets are commercially available. A guide wire is advanced into the main portal vein. The tract between the hepatic and the portal vein is dilated with an angioplasty balloon catheter (8–10 mm) (Figure 2) followed by stent placement to maintain the communication between both vessels patent (Figure 3). Various TIPS stents can be used (bare stents and PTFE-covered stents). The portacaval gradient after TIPS must be be lower than 12 mmHg (the cut-off level associated with complications of portal hypertension) [1, 9].

This technique is now well standardized in specialized centers. The use of ultrasound or transhepatic portography can help localize the intrahepatic portal vein, particularly when anatomic variants or marked liver distortion is observed particularly in cirrhotic patients [8, 10]. Over the years, PTFE-covered stents have replaced bare stents as they markedly improved the long-term patency of the shunt and also prevent portobiliary fistulae [11–13]. The metallic stent should be placed near the junction between the hepatic vein and the vena cava and no more than 1-2 cm below the bifurcation of right and left portal veins. Moreover, the covered part of the stent should not be inside the portal vein as it can block the retrograde intrahepatic portal flow, which may result in intrahepatic portal vein thrombosis. Provided that these principles are followed, liver transplantation can be performed safely without interference of the stent at the time of portal vein and vena cava clamping [14, 15].

## 3. Contraindications

Contraindications are summarized in Table 1. As mentioned previously, portal hypertension is associated with a hyperdynamic circulation (increased cardiac output, increased splanchnic blood flow and decreased systemic resistances). Hemodynamic changes induced by TIPS are spectacular, with a sudden increase in the cardiac output secondary to diversion of splanchnic blood flow into the systemic circulation [16, 17]. Therefore, any impairment in the right ventricle function before TIPS is a problem, as congestive liver failure may be observed after TIPS-induced increase in cardiac output. An evaluation of cardiac function is required before TIPS. On the other hand, even if the hyperdynamic circulation worsens after the procedure, this phenomenon is often transient [16]. The other contraindications are quite obvious. Pre-TIPS chronic recurrent disabling hepatic encephalopathy (HE) is an absolute contraindication, but the onset of an episode of HE induced by precipitants (such as bleeding, sepsis, electrolyte imbalance) before TIPS does not preclude the use of this procedure. The presence of portal vein cavernoma or portal vein thrombosis is no longer an absolute contraindication and may even become an indication as technical advances allow recanalization of the portal vein in some selected cases [18–21]. A transhepatic or a transplenic approach can be helpful to catheterize the main portal vein and facilitates the TIPS procedure.

Many prognostic studies have been published for the prediction of short-term survival after TIPS [22–24]. It is now well recognized that a Pugh score higher than 12 most often represents a contraindication as multiorgan failure occurs in a vast majority of these cases after TIPS [6]. The Meld score has been initially validated as the best predictor of the 3 months survival rate [25–27]. However, TIPS may be performed as a temporary hemostatic measure in a patient already placed on the waiting list for liver transplant.

## 4. Complications

They are summarized in Tables 2 and 3. Comparison of complication frequency is difficult to evaluate in the literature due to the patient characteristics, the expertise of the center and the study period [28, 29].

### 4.1. Acute Complications

Acute complications might occur during TIPS placement or within hours or days after the procedure and include neck hematoma, arrhythmia, stent displacement, hemolysis, bilhemia, and shunt thrombosis. Neck hematoma can be prevented by haematological preparation and ultrasound-guided puncture of the jugular vein. Arrhythmia may occur but is self-limited when the distal tip of the guide wire is removed from the right atrium. Bilhemia results from a fistula between a biliary radicle and the portal vein. It must be suspected when a sudden rise of direct bilirubin occurs without any symptoms. It can be proven by shuntography or ERCP and treated by a covered stent across the fistula [30, 31]. Hemolysis is transient and is related to the fragmentation of red blood cells in the metallic stent before endothelialization [32, 33]. The obstruction of a small hepatic vein by a PTFE-covered stent may induce a “segmental” Budd Chiari syndrome with a transient increase in serum bilirubin and transaminases. This phenomenon is self-limited in a majority of the cases [34, 35]. Acute shunt thrombosis (less than 5%) is rare and it is usually due to a portobiliary fistula or in some cases to stent malfunction [36, 37]. The usefulness of phenprocoumon to prevent stent thrombosis is not well established [38]. The shunt can be recanalized but at the same time the fistula must be closed with a covered stent.

Life-threatening complications are very rare (less than 1%) and include hemoperitoneum, hemobilia, liver ischemia, cardiac failure, and sepsis [28]. Hemoperitoneum is most often related to a puncture of the liver capsule; it is usually self-limited. A dissection of the portal vein in its extrahepatic part is life threatening and can be treated with a covered stent. Hemobilia results from a procedure-related fistula between a hepatic artery and the biliary tract. It is treated by embolization. Liver ischemia may follow an accidental catheterization of an intrahepatic artery followed by its thrombosis [39, 40]. Cardiac failure is due to a rapid increase in cardiac output; it may be severe and diuretics can be tried but, in life threatening cases, the obstruction of the shunt may be needed. Finally, sepsis is a potential complication, but antibioprophylaxis can prevent it in a vast majority of cases.

### 4.2. Chronic Complications

Chronic complications are more frequent and their management may be difficult. Congestive heart failure is related to a high cardiac output following TIPS. Clinically the patients develop sodium retention and right sided heart failure; in severe cases, treatment with diuretics and vasodilators does not work and obstruction of the shunt may be necessary. Portal vein thrombosis is very rare. It occurs more often when the stent is not correctly placed inside the portal or the hepatic vein, thus obstructing the shunt flow [8]. It may be observed in patients with a hypercoagulable state and in this situation life-long anticoagulation is needed. As observed after surgical portacaval shunt, progressive liver failure may follow TIPS implantation. The first sign is a progressive increase in the serum bilirubin, which is then followed by a rise in INR, onset of encephalopathy, and death due to multiorgan failure within weeks after TIPS. Even if poor pre-TIPS liver function is a risk factor, some patients with a good hepatic reserve may also develop this serious complication after TIPS. Liver transplantation is the only option in this situation.

TIPS is a portacaval shunt; therefore, not surprisingly post-TIPS HE remains a problem. HE episodes are observed in 30–40% of cirrhotic patients, and as opposed to that observed in patients without TIPS, no precipitant can be identified in a majority of cases.

Chronic recurrent disabling HE can occur in 5–10% and may lead to a complete loss of the patient's autonomy. Several pre-TIPS parameters have been tested to predict post-TIPS hepatic encephalopathy (Table 4). Age, pre-TIPS encephalopathy, and the Pugh score are probably the most useful predictors [41–49]. Prophylaxis with lactulose is not useful [50]. The medical management is difficult and in many cases the only option is to reduce the diameter of the stent or preferably to occlude it [51]. HE clears quickly after the obstruction, but portal hypertension recurs with its associated potential complications (ascites and variceal bleeding). Embolization of varices before TIPS occlusion might be useful measure to prevent variceal rebleeding.

**Figure 4:**
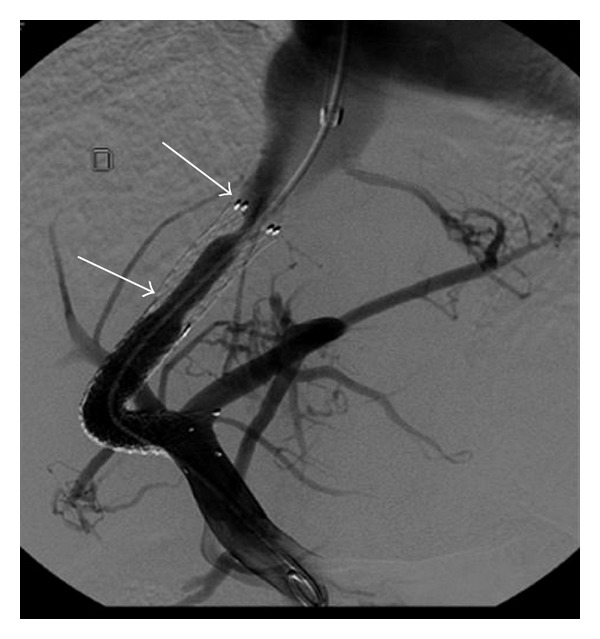
Portography in patient treated one year ago with a TIPS made of a combination of one PTFE-covered stent and one bare stent. Pseudointimal hyperplasia developed only on the bare part of the TIPS (arrows) and induced TIPS dysfunction.

The function of the TIPS is usually evaluated using Doppler ultrasonography. The direction of intrahepatic portal flow, the flow volume in the stent, and the presence of increased velocity in the stent are useful criteria to detect shunt dysfunction and to decide if a shunt revision is needed with an angiographic intervention [52, 53]. However, the sensitivity and specificity of this modality are only 80–85%. Shunt dysfunction results from an intimal hyperplasia in the stent [54] and is more frequent in the hepatic vein part of the shunt (Figure 4). This phenomenon was observed at 1 year in nearly 80% of cases treated with bare stents and could not be prevented with acetyl salicylic acid [55] or trapidil + ticlopidine [56]. When PTFE-coated stents are used the one-year rate of shunt stenosis is only 10–15% [12] (Figure 3). Treatment includes dilatation of the stenoses and/or implantation of a new covered stent in this area. TIPS involves a foreign material chronically implanted in the liver, and cirrhotic patients are often immunocompromised and therefore susceptible to infection. But, surprisingly, the infection of the stent (the so-called TIPSitis) is exceptional [5]. Diagnostic criteria include repeated episodes of septicaemia without any other detectable source of infection. It is best treated with long-term antibiotherapy [57, 58].

## 5. Indications

TIPS has been used to treat many complications related to portal hypertension. The relative efficacy of TIPS has been tested with randomized controlled trials, (refractory ascites, variceal bleeding), whereas other indications have been evaluated in uncontrolled case series.

### 5.1. Gastrointestinal Bleeding

#### 5.1.1. Oesophageal Variceal Bleeding

**Primary Prophylaxis**

Bleeding from oesophageal varices is a common and severe complication of portal hypertension. Prevention of the initial bleeding can be achieved in a number of cases by endoscopic variceal ligation or *β*-blocker treatment. However, TIPS has never been tested in this situation as previous experience with surgical portacaval shunts has clearly demonstrated that this approach is associated with higher morbidity and mortality rates [60].

#### 5.1.2. Acute Bleeding Episode

When an initial bleeding occurs, it is usually controlled with less invasive endoscopic treatment and/or pharmacological therapy. In rare cases bleeding remains uncontrollable, and TIPS has been used as a rescue treatment with good results for bleeding control. However, prognosis relies on the general condition of the patient, the value of the liver function reserve, and the associated comorbidities [61–64]. However, a recent randomized controlled trial evaluated the use of emergent TIPS as compared to standard medical therapy in patients with severe portal hypertension and a Pugh score of 7 to 13 [65]. Treatment failure was more frequent in the medical group (50% versus 12%) and the survival rate was better in the TIPS group (11 versus 38%). This approach could justify the use of TIPS early after bleeding episodes in patients with moderate or severe liver failure and severe portal hypertension. These promising results are in line with that observed in a case series of cirrhotic patients Child A or B who underwent emergency portacaval shunt surgery [66] but should be confirmed by other controlled trials.

#### 5.1.3. Secondary Prophylaxis

Bleeding tends to recur frequently after a first episode. *β*-blockers and variceal band ligation have both been demonstrated to lower the incidence of rebleeding [1]. TIPS has been tested against these two modalities in several prospective controlled trials [67–78]. Meta-analyses have demonstrated that TIPS was more efficient in preventing rebleeding but it was more frequently followed by episodes of encephalopathy, and survival was not different between groups [59, 79, 80] (Table 5). TIPS has also been compared with surgical shunts or oesophageal transaction [81–83], but results are difficult to interpret because all the patients were good operative risks, and the studies were performed before the introduction of PTFE-coated stents. Therefore, TIPS is not recommended as a first-line therapy for secondary prophylaxis of variceal bleeding.

#### 5.1.4. Gastric Variceal Bleeding

**Figure 5:**
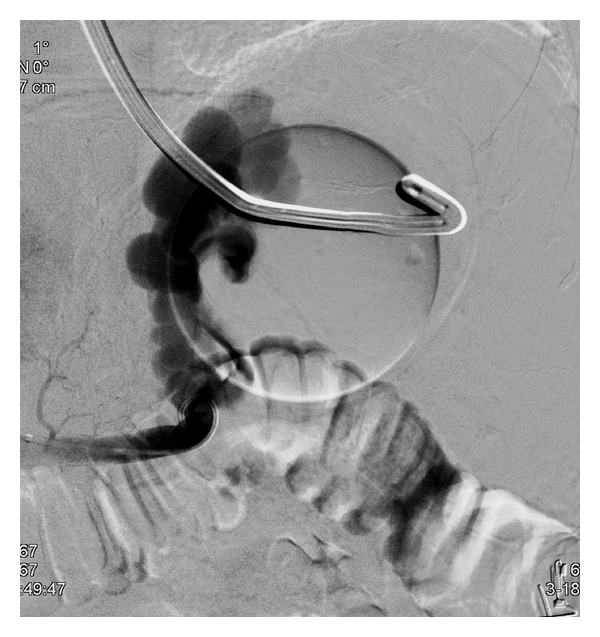
Transjugular portography in a patient bleeding from gastric varices. Note that balloon tamponade did not suppress fundal varices filling.

**Figure 6:**
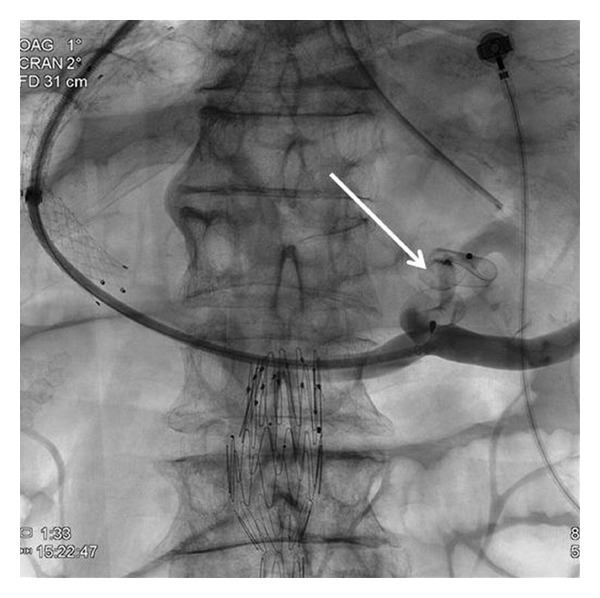
Embolisation of fundal varices using an Amplatzer® vascular plug, in conjunction with the TIPS procedure due to persistence of fundal varices filling despite a functional TIPS.

Bleeding from gastric varices is often severe and difficult to control, particularly when fundal varices are involved. The first-line treatment is endoscopic sclerotherapy with cyanoacrylate [84]. TIPS has been used in a number of uncontrolled trials in patients in whom endoscopic therapy failed [85, 86]. A recent controlled trial has shown that TIPS is more efficient than cyanoacrylate in prevention of rebleeding (secondary prophylaxis) from large gastric varices [87]. This interesting finding must be confirmed by other groups and after a long-term followup. It should be mentioned that due to the large size of fundal varices, the risk of rupture is still present even at a low portacaval gradient (<12 mmHg) after TIPS [88, 89]. This is probably best explained by the relationship between the variceal tension (and therefore the risk of rupture) and the variceal size. For this reason, it is now recommended to embolize gastric varices at the time of TIPS placement [90] (Figures 5 and 6).

#### 5.1.5. Ectopic Varices

**Figure 7:**
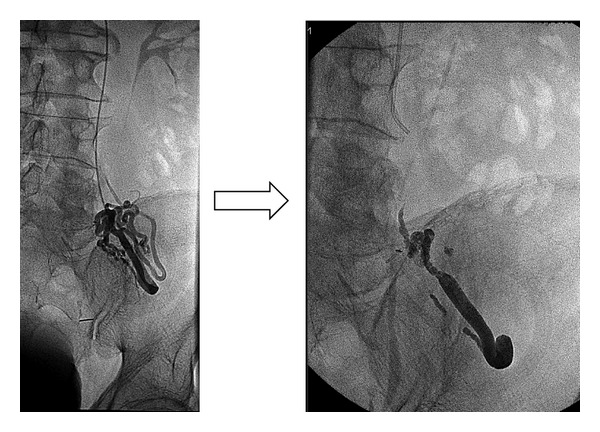
(a) Stomal varices in a patient with cirrhosis and colostomy. (b) Treatment of stomal varices with TIPS and embolization using histoacryl injections.

Varices may develop anywhere along the digestive tract in patients with portal hypertension (duodenum, jejunum, colon, rectum, stomies) and may bleed. Local treatments are either impossible or associated with a high rate of rebleeding. The best approach is the TIPS procedure, which can be combined with embolization of the varices [91, 92] (Figure 7).

#### 5.1.6. Portal Hypertensive Gastropathy

These gastric lesions rarely induce problematic bleeding. Anecdotal case reports suggest that TIPS may control bleeding in these patients [93].

#### 5.1.7. Gastric Antral Vascular Ectasia (GAVE)

Chronic bleeding from GAVE may be difficult to manage. However, TIPS does not help to control haemorrhage, probably because these vascular lesions are related to liver disease and not to portal hypertension [93–95].

### 5.2. Ascites

Ascites is a frequent complication of portal hypertension. It may become resistant to medical treatment in nearly 5–10% of cases [97], and the TIPS procedure has been evaluated for this situation in case series [98–100] and several prospective randomized controlled trials [101–106]. TIPS-induced decrease in portal pressure leads to a good control of ascites in a majority of cases and more often than repeated large volume paracentesis. However, hepatic encephalopathy is observed more frequently, and survival is not improved in a majority of trials [96, 107, 108] (Table 6). However, a recent meta-analysis showed different results after analysing individual data [109]. Moreover, a recent study demonstrated that survival was better in the TIPS group as compared to the paracentesis group; it should be mentioned that in this study, the patients had good liver and renal function [103]. Therefore, this issue is still controversial. There is no clinical controlled trial on the long-term efficacy of PTFE-covered stents in the treatment of refractory ascites. It is now agreed that TIPS may be offered to cirrhotic patients with moderately impaired liver function, without organic kidney disease and preferably in younger patients (less than 65 years) [1]. Liver transplantation should be considered as a backup in case of TIPS failure [110]. The quality of life must be also be considered in the decision making process [111] if transplantation is not an option.

### 5.3. Pleural Effusion

 This is an equivalent of ascites, but the tolerance is poor as only a limited amount of fluid in the pleural may induce disabling dyspnea. Repeated pleuracentesis is risky and chronic drainage is often associated with infection of the fluid. TIPS is a good option, but the risks of severe hepatic encephalopathy and/or liver failure following TIPS are similar to that observed in ascitic patients [112–114].

### 5.4. Hepatorenal Syndrome

The chronic form of functional renal failure associated with ascites (hepatorenal syndrome type 2) is usually reversible after TIPS; by contrast, hepatorenal syndrome type 1 which is progressive, more severe, and associated with progressive liver failure usually responds less well as TIPS may aggravate the liver insufficiency [115–117]. It has no role in these patients except for highly selected cases as a bridge to liver transplantation.

### 5.5. Budd Chiari Syndrome

**Figure 8:**
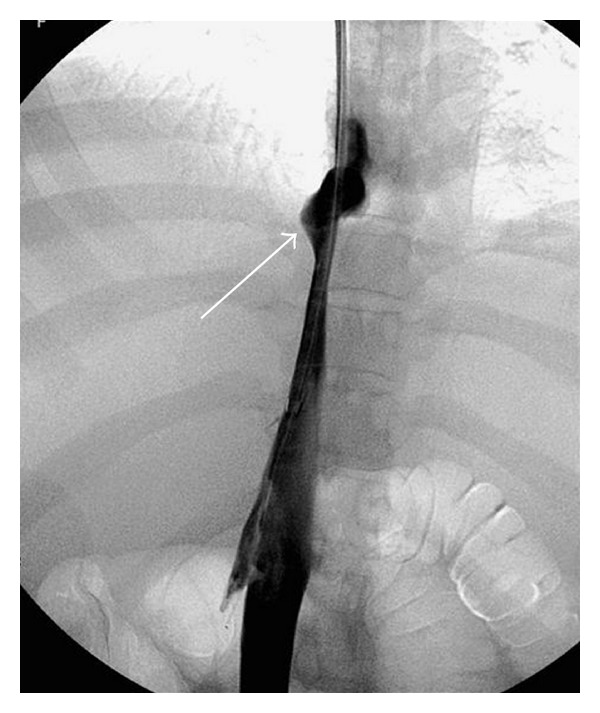
Cavography in a patient with Budd Chiari syndrome. Note that the right hepatic vein was almost completely occluded (arrow).

**Figure 9:**
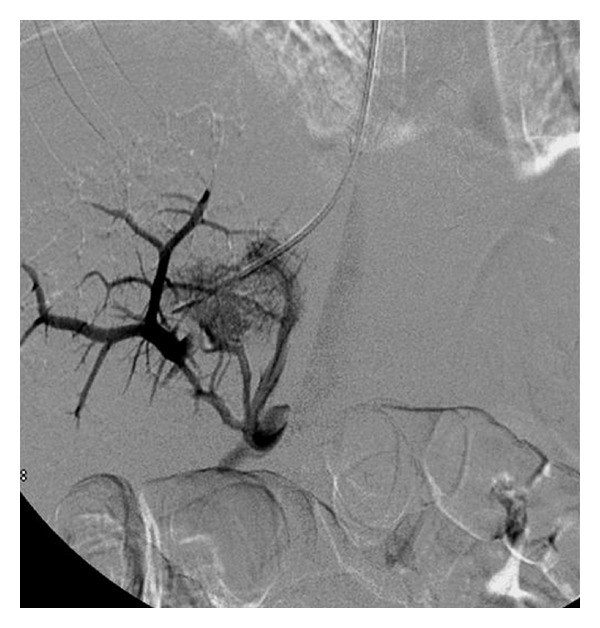
Transjugular portography in the same patient.

**Figure 10:**
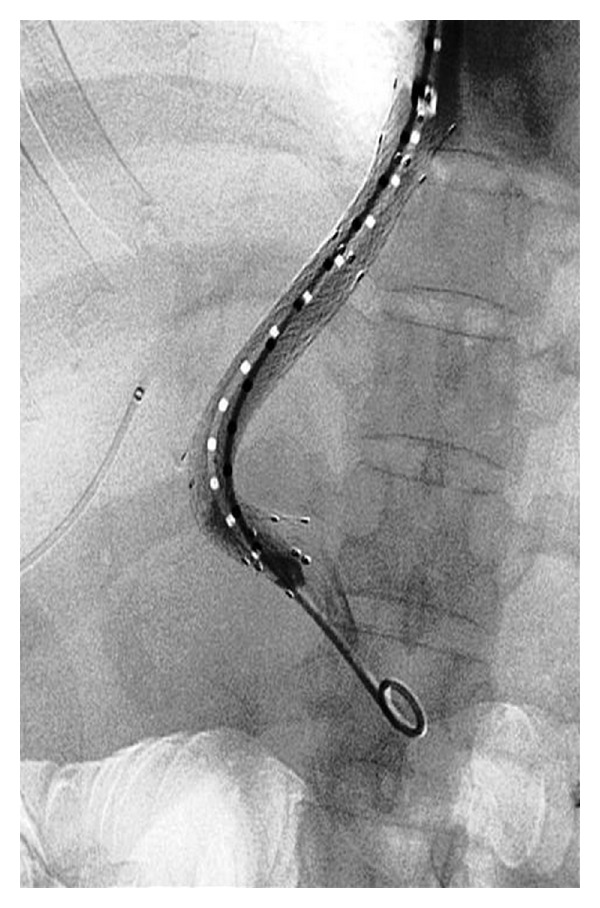
Successful TIPS placement after portal vein catheterization by a transjugular approach.

The management of this syndrome includes diuretic therapy and chronic anticoagulotherapy. In refractory cases, surgical side-to-side portacaval shunt has been used in the past but is no longer used due to the operative risks and the conflicting results [118]. TIPS, which is a nonsurgical equivalent, has been widely tested and demonstrated promising results (control of ascites, reversal of liver failure) in large series [119, 120]; however, the technique of TIPS placement is difficult given the absence of hepatic veins and the caudate lobe hypertrophy (Figures 8, 9 and 10). These patients must be anticoagulated life long. There is no controlled trial comparing TIPS with liver transplantation, but the good results observed after TIPS justify its use first, transplantation being considered in TIPS failure.

### 5.6. Veno-Occlusive Disease

Several case reports have evaluated the TIPS procedure in the treatment of veno-occlusive disease with some good results [121, 122].

### 5.7. Miscellaneous Indications

#### 5.7.1. Preoperative TIPS

It has been suggested that relief of portal hypertension before abdominal surgery in cirrhotic patients could decrease the perioperative bleeding and postoperative complications, such as, ascitic leak [123]. However, TIPS-associated complications are not infrequent [124]; the best candidates for preoperative TIPS are cirrhotic patients with well-preserved or moderately impaired liver function (Pugh class A or B) and a significant amount of venous collaterals in the operative area. It should also be mentioned that preoperative TIPS would prevent the formation of stomal varices after surgery, which often induce recurrent bleeding (Figure 7).

#### 5.7.2. Hepatopulmonary Syndrome

A recent review reports 6 cases of hepatopulmonary syndrome with an improvement in oxygenation after TIPS placement in 5 patients [125–127]. The rationale of this approach is difficult to understand as worsening of vasodilatation usually follows the TIPS procedure, which could aggravate hypoxemia. Therefore, the mechanism of action is unknown.

## 6. The TIPS Unit

Experience with this procedure over last 20 years clearly demonstrates the need for a multidisciplinary approach. First of all, the indications for TIPS should be discussed rigorously according to a risk benefit approach; preoperative evaluation should include not only the liver function parameters, the cardiac function, but also the assessment of the comorbidities and the evaluation of the risks of post-TIPS chronic encephalopathy. The benefits of TIPS implantation must be weighed against that of liver transplantation. Therefore, hepatologists (or gastroenterologists), cardiologists, interventional radiologists, intensive care specialists, and transplant surgeons play a role in the decision making process. Primary patency higher than 90% after the TIPS placement is a prerequisite in such a TIPS unit. Followup is also crucial as post-TIPS complications may occur and must be treated. Ideally, these patients must be followed regularly in a specialized TIPS clinic, and the surveillance of the TIPS function as well as screening for hepatocarcinoma is mandatory. The collaboration of a highly trained nurse is essential.

## 7. Conclusions

The TIPS procedure is now a well-established treatment of complications of portal hypertension. Technical advances and well-designed clinical studies provide a scientific basis to define the best indications. Cost effectiveness analysis must be done in the future taking into account recent developments (technical improvements, better selection of patients, and better management after TIPS). However, severe complications still exist and have to be addressed as stated in a recent editorial [128].

## Figures and Tables

**Table 1:** Contraindications for the TIPS procedure.

Absolute
(i) Right sided heart failure
(ii) Biliary tract obstruction
(iii) Uncontrolled infection
(iv) Pulmonary hypertension
(v) Chronic recurrent disabling hepatic encephalopathy
(vi) Hepatocellular carcinoma involving hepatic veins

Relative
(i) Severe liver failure (Pugh score >12)
(ii) Portal vein thrombosis
(iii) Multiple hepatic cysts

**Table 2:** Acute complications after TIPS placement.

Minor or moderate
(i) Neck hematoma
(ii) Arrhythmia
(iii) Stent displacement
(iv) Hemolysis
(v) Bilhemia
(vi) Hepatic vein obstruction
(vii) Shunt thrombosis
life threatening
(i) Hemoperitoneum
(ii) Hemobilia
(iii) Liver ischemia
(iv) Cardiac failure
(v) Sepsis

**Table 3:** Chronic complications after TIPS placement.

(i) Congestive heart failure
(ii) Portal vein thrombosis
(iii) Progressive liver failure
(iv) Chronic recurrent encephalopathy
(v) Stent dysfunction
(vi) “TIPSitis”

**Table 4:** Risk factors for post-TIPS encephalopathy.

Age
Gender
Etiology
HE before TIPS
Child-Pugh score
Portohepatic gradient
Shunt diameter
Creatinine
Indication

**Table 5:** Comparison of TIPS and endoscopic and/or pharmacological therapy in the prevention of oesophageal variceal rebleeding (from Zheng et al. [59]).

Treatment	Number of patients	Rebleeding rate *n* (%)	Encephalopathy *n* (%)	Mortality *n* (%)
TIPS	440	86 (19)	148 (33)	111 (26)
Sclerotherapy /pharmacological therapy	443	194 (44)	86 (19)	98 (22)

**Table 6:** Comparison of TIPS and large volume paracentesis in the treatment of refractory ascites (from D'Amico et al. [96]).

Treatment	Number of patients	Recurrence of ascites *n* (%)	Encephalopathy *n* (%)	Mortality *n* (%)
TIPS	149	66 (44%)	72 (48%)	69 (46%)
Paracentesis	156	135 (87%)	51 (33%)	82 (54%)
